# Barriers to Physical Activity in Low-Income Older Adults Living in Senior Housing

**DOI:** 10.3390/healthcare13101159

**Published:** 2025-05-16

**Authors:** Carolyn Kalata, Ramon Reyes, Kamal Kuhail, Janet L. Larson, Weiyun Chen

**Affiliations:** 1 School of Kinesiology, University of Michigan, Ann Arbor, MI 48109, USA; ckalata@umich.edu (C.K.); ramonrey@umich.edu (R.R.); kkuhail@umich.edu (K.K.); 2 School of Nursing, University of Michigan, Ann Arbor, MI 48109, USA; janetlar@umich.edu

**Keywords:** low-income, older adults, physical activity, barriers

## Abstract

While a majority of older adults fail to engage in recommended levels of physical activity (PA), lower-income older adults face unique challenges. They are at greater risk for low levels of PA, but little is known about the barriers they face. Objectives: This study aimed to investigate self-reported PA and barriers to PA for older adult residents of subsidized low-income senior housing, comparing barriers for those with lower and higher levels of PA. Methods: Ninety-two older adults (mean age 76.57 (SD = 7.50)) were recruited from low-income senior housing facilities. They completed a demographic questionnaire, the International Physical Activity Questionnaire (IPAQ), which measures MET-minutes/week of engaging in vigorous, moderate, and light PA levels, and the 27-item Inventory of Physical Activity Barriers (IPAB), which measures multifaceted barriers to PA. Results: The most common barrier for all residents was the PA priority. Independent *t*-tests revealed that the low-active group faced greater barriers than the high-active group in physical health barriers (*t* = 2.329, *p* = 0.022), PA priority of (*t* = 2.836, *p* = 0.006), environmental barriers (*t* = 2.072, *p* = 0.042), and total PA barriers (*t* = 2.281, *p* = 0.025). No significant differences were found between the low-active and high-active groups for emotional health barriers, skill barriers, external factor barriers, and social barriers. Conclusions: The low-active older adults were less likely to overcome barriers, such as physical health issues, PA priority, and environmental factors. Further research is necessary to gain a deeper understanding of the characteristics and underlying mechanisms of these barriers and to develop effective strategies for addressing them. However, findings should be interpreted cautiously due to the small and convenient sample and potential bias from self-reported PA measures.

## 1. Introduction

Older adults are less active than other segments of the population, which negatively affects their health, physical functioning, quality of life, and longevity. Physical activity (PA) levels are particularly low for low-income older adults [1]. Low-income older adults are at greater risk for poor health and functioning and inadequate social and environmental resources for PA engagement, but this is an understudied population, and little is known about the barriers they face. The larger body of research indicates that multiple barriers interact at the individual, social, and environmental levels.

The general population of older adults faces multiple barriers that limit their engagement in PA and contribute to a sedentary lifestyle. Two recent systematic reviews identify barriers to PA engagement for older adults from the general population [2,3]. The most reported barriers were physical health and functioning, lack of motivation, fear of falling, and environmental barriers [3]. Similar barriers identified by qualitative research included the individual’s capability and perceived risk of injury [2]. Other barriers include, but are not limited to, a lack of social support, lack of knowledge, and emotional factors.

Low-income older adults experience additional barriers that interact and make it more challenging to engage in PA. At the individual level, lower socioeconomic status is associated with lower levels of functioning physically, cognitively, and mentally [4]. Older adults with low functional skills have more difficulty performing activities of daily living and participating in PA [5]. Physically inactive older adults have significantly lower fitness expectations than those who are physically active and may be intimidated by fitness facilities [4], especially if they have not had prior exposure during their youth. Low health literacy is a potential problem in low-income communities, and low-income older adults may not be aware of the benefits of physical activity [6]. Low income and poverty are also associated with stress, which can lead to physical and mental health problems. People are less likely to prioritize physical activity if they are stressed by financial issues.

Social barriers to PA for low-income older adults include limited social support for physical activity and limited knowledge about its benefits. Smaller social networks, a lack of transportation, and affordability negatively affect PA engagement. A systematic review of 27 articles reported that older adults who had greater social support for PA from their family members were more likely to engage in leisure-time PA [7]. Having a companion for PA is important; however, this can be problematic for older low-income adults if they become socially isolated.

Neighborhood socioeconomic disadvantage is associated with less participation in various forms of PA [8]. The financial constraints of lower-income neighborhoods can restrict access to resources that support PA, including safe spaces for exercise, walking paths, and street lighting. Community barriers include a lack of recreational facilities and inadequate public transportation.

In general, low-income older adults face substantial barriers to establishing and maintaining engagement in PA, but the impact on PA is less clear. Living in senior housing could mitigate some of the commonly identified barriers, including safe spaces and social opportunities for PA. The purpose of this research was to investigate self-reported PA and barriers to PA for older adult residents of low-income senior housing, comparing barriers for those with lower and higher levels of PA. We hypothesized that residents with lower PA will have more barriers to PA than those with higher PA. The identified barriers to PA can be used as targets for interventions to increase PA in this population.

## 2. Methods

### 2.1. Study Design

This study used a cross-sectional study design. Accordingly, an anonymous survey consisting of self-reported physical activity and barriers to physical activity questionnaires was collected from voluntary participants.

### 2.2. Participants

Participants were recruited from 10 low-income senior housing in surrounding cities, identified as being low-income by the Michigan Housing Bureau. This included subsidized housing for residents with a median income less than 50% of the median income of the area. Recruitment efforts included: (1) mailing or emailing site managers or service coordinators the study invitation letter, explaining the study purpose, protocols, and participants’ involvement, or calling site managers or service coordinators if their email address was not available; (2) site manager or service coordinator helped post recruitment flyers containing a survey QR code on bulletin boards and verbally informing residents about the study; and (3) with the site manager or the service coordinator’s approval, we distributed the flyer to each resident’s mailbox in order to reach every resident and provide the study opportunity for all residents. Then, paper copies of the study were distributed to senior housing sites where this method of survey was preferred, and the principal investigator provided informational meetings to present further details about the study. On the first page of the survey, the study purpose, voluntary and confidential nature of the study, time estimated to complete the survey overall, inclusion criteria for the study, and date and where to return their completed survey are provided. According to the first purpose of this study—to investigate self-reported PA and barriers to PA for older adult residents of low-income senior housing—individuals were eligible to participate in this study if they met the following inclusion criteria and none of the exclusion criteria. The criteria included: (1) aged 65 years or older, (2) living in low-income senior housing, and (3) voluntarily agreeing to participate in this study. Exclusion criteria included: (1) having been diagnosed with cognitive impairment such as Alzheimer’s disease and related dementias; (2) a diagnosis of severe depressive symptoms; and (3) having severe obesity, defined as a BMI of 40 or higher. Participants could either complete paper copies or online versions of the survey depending on their preference. The study protocol was reviewed and approved by the University of Michigan, Institutional Review Board [HUM00240091], and participants signed a consent form prior to completing the surveys.

### 2.3. Measures

Barriers to PA. The 27-item Inventory of PA Barriers (IPAB) was designed by Wingood et al. [9] to measure the frequency of multilevel factors that prevent individuals from engaging in physical activity. It was designed to be used by clinicians in the clinical setting to identify targets for intervention. The IPAB consists of seven sub-scales (factors) and one stand-alone item. They are (1) environmental barriers such as accessibility or affordability of physical activity (six items); (2) physical health barriers such as balance or poor health (five items); (3) PA priority barriers such as attitudes and importance of PA (four items); (4) emotional health barriers such as anxiety or depression (four items); (5) external factor barriers such as time or responsibilities (three items); (6) skill barriers such as competence (two items); and (7) social barriers in terms of a support system and having others to be physically active with (two items). The stand-alone item was energy. Participants respond to each item on a Likert 5-point rating scale, with a minimum score of 1 and a maximum score of 5 (1 = Never, 2 = Rarely, 3 = Sometimes, 4 = Often, and 5 = Always). Item scores for each subscale were averaged to obtain a mean score; higher scores indicate a greater number of barriers and vice versa. The IPAB has displayed high test–retest reliability (intraclass correlation coefficient = 0.99), high construct validity, and acceptable internal consistency (Cronbach’s α = 0.91 for the total scale) [9]. Cronbach’s α for this survey was 0.93.

PA. The 7-item International PA Questionnaire (IPAQ) was designed by an International Consensus Group to measure the volume of PA [10]. Participants report the number of minutes per day and days per week they engaged in vigorous PA (VPA), moderate PA (MPA), walking, and the number of hours per day spent sitting in the past week. Using these data, MET-minutes/week were calculated by multiplying the intensity factor by the number of minutes and days reported. The intensity factor was 8.0 for VPA, 4.0 for MPA, and 3.3 for walking. Total PA MET-minutes/week was calculated by adding VPA MET-minutes/week, MPA MET-minutes/week, and walking MET-minutes/week. The IPAQ is widely used as a self-report measure of PA, and its validity has been supported by research across 12 countries [10]. The Cronbach α for this survey was 0.72.

### 2.4. Data Collection

The survey was expected to take 8–10 min to complete based on timing during pilot testing. Completed paper surveys were returned to a dropbox in front of the main office. Participants were asked to answer each question as honestly and thoughtfully as possible. They were also assured that there were no wrong answers. As an incentive, a USD 15 Amazon gift card was raffled off to those who participated in the study.

### 2.5. Statistical Analysis

Descriptive statistics were calculated for all variables, frequency, and percentage for categorical variables, and mean and standard deviation for continuous variables. Participants were classified as minimally active or moderately active based on the median MET-minutes/week (706.5 MET-minutes/week). Low-active was defined as ≤706 MET-minutes/week and high-active as >706 MET-minutes/week. Groups were compared using independent sample *t*-tests for each variable. The level of significance was set at 0.05. All data were analyzed using the IBM SPSS 28 version.

## 3. Results

A total of 92 older adults participated in this study, comprising 69 women and 23 men, with a mean age of 76.57 years (7.50). The low-active group had a mean age of 76.36 years (7.904), while the high-active group had a mean age of 76.76 years (7.184). A description of the participants is presented in Table 1. The low-active group and high-active group consisted of 46 participants each. The two groups had similar gender distributions (low-active: 26.1% male, 73.9% female; high-active: 23.9% male, 76.1% female) and racial compositions (white: low-active 67.4%, high-active 65.2%); 32% and 30% were from other racial or ethnic backgrounds.

As presented in Table 2, the total PA MET-minutes/week for the total sample was 1711.13; for the low-active group, it was 193.18; and for the high-active group, it was 3229.08. The low-active group reported an average of 15.3 MET-minutes of VPA, 47.7 MET-minutes of MPA, and 130.1 MET-minutes of walking each week. The high-active group reported 561.2 MET-minutes of VPA, 806.5 MET-minutes of MPA, and 1861.3 MET-minutes of walking each week (See Table 2). The low-active group scored significantly lower than the high-active group in time spent engaging in VPA (*t* = 4.352, *p* < 0.001, *Cohen’s d* = 0.907), MPA (*t* = 5.250, *p* < 0.001, *Cohen’s d* = 1.095), walking (*t* = 2.029, *p* = 0.045, *Cohen’s d* = 0.423), and total PA (*t* = 3.426, *p* = 0.001, *Cohen’s d* = 0.714).

Barriers to PA are presented in Table 2. The IPAB total score for the full sample was a mean of 2.14 (0.73). According to the IPAB scoring criteria, scores range from a minimum of 1 to a maximum of 5, with lower scores indicating fewer barriers to PA. The low-active group self-reported “sometimes” experiencing barriers related to physical health (mean = 2.50, SD = 1.05) and PA priority (mean = 2.66, SD = 1.04), and “rarely” experiencing barriers due to environmental factors (mean = 2.02, SD = 1.07). Overall, they reported barriers to PA close to “sometimes”. In contrast, the high-active group self-reported “rarely” facing barriers related to physical health (mean = 2.04, SD = 0.83) and exercise prioritization (mean = 2.09, SD = 0.89), and “almost never” experiencing environmental barriers (mean = 1.64, SD = 0.63). Overall, their reported barriers to PA were less frequent than “rarely”.

Independent sample *t*-tests revealed the low-active group experienced significant barriers in physical health (*t* = 2.329, *p* = 0.022, *Cohen’s d* = 0.486), PA priority (*t* = 2.836, *p* = 0.006, *Cohen’s d* = 0.591), environmental factors (*t* = 2.072, *p* = 0.042, *Cohen’s d* = 0.432), and total PA barriers (*t* = 2.281, *p* = 0.025, *Cohen’s d* = 0.476), compared to their counterparts in the high-active group. In contrast, no significant differences were found between the low-active and high-active groups for emotional health barriers (*t* = 1.505, *p* = 0.136, *Cohen’s d* = 0.314), skill barriers (*t* = 0.834, *p* = 0.407, *Cohen’s d* = 0.174), external factor barriers (*t* = 1.216, *p* = 0.227, *Cohen’s d* = 0.254), and social barriers (*t* = 1.416, *p* = 0.160, *Cohen’s d* = 0.295).

Among health variables, the low-active group was significantly less likely to walk six blocks (*t* = −2.631, *p* = 0.010, *Cohen’s d* = 0.557), compared to the high-active group. No significant differences were found between the two groups in the likelihood of leaving the house (*t* = 0.704, *p* = 0.484, *Cohen’s d* = 0.147) and having a fall (*t* = 0.426, *p* = 0.671, *Cohen’s d* = 0.089).

## 4. Discussion

Older residents of low-income senior housing reported a wide range of PA; the low-active residents were inactive, and the high-active residents were moderately active. Residents with lower PA reported significantly more barriers to PA than people with higher PA. They reported more frequently experiencing total barriers and more physical health, environmental, and PA priority barriers, compared to their counterparts in the high-active group. However, it is important to note that residents in both low-active and high-active groups reported they rarely experienced emotional health, skills, social, and external barriers to PA. Residents with low PA reported significantly less time spent in all types of PA: moderate and vigorous PA, and walking. The barriers reported by low-income residents of senior housing will enable programs to intervene by targeting the most relevant issues.

Our findings are consistent with the observation that physically inactive older adults have more perceived barriers to regular PA [11]. However, low-income senior housing residents in this study reported a greater number of barriers compared to prior research with mixed-income community-dwelling older adults [9]. Wingood et al. [9] reported a mean IBPA score of 1.8 (SD = 0.5). Participants with <150 min/week of MVPA reported more barriers, IBPA score of 2.1 (SD = 0.4), whereas those with ≥150 min/week of MVPA reported fewer barriers, 1.6 (SD = 0.4). The differences between the two studies could be related to the lower income of older adults in our study.

The most common barrier for all residents was prioritizing PA but low-active residents reported significantly greater problems with prioritizing. This is consistent with prior research in older adults. A recent systematic review found that motivation was one of the most reported barriers to older adults’ participation in intergenerational physical activity programs [12]. Prioritizing physical activity is a complex issue that likely interacts with other barriers. For example, when older adults have lower expectations for PA, they may be less likely to make the time for PA [11].

Physical health problems were associated with lower levels of PA and the second most common barrier for low-active residents. The frequency of physical health-related barriers is consistent with prior research in other older adult populations. Physical health barriers commonly include chronic diseases such as osteoarthritis, cardiovascular disease [13], limited mobility, and cognitive decline. The fear of exacerbating a chronic condition may discourage older adults from engaging in PA. In addition, older adults with severely limited mobility report more barriers, including poorer health, fear and negative experiences, lack of an exercise companion, and an unsuitable environment for exercise compared to those with no mobility limitation [14].

Environmental issues were the least common barriers for both groups of residents, but they were significantly greater for the low-active residents. Prior research produced inconsistent results with respect to the effects of the neighborhood environment on the PA of older adults. Chudyk and colleagues [15] studied low-income older adults and found no relationship between the neighborhood’s walkability and objectively measured PA. In contrast, Barnett et al. reported that safe, walkable, and esthetically pleasing neighborhoods with access to overall and specific destinations and services positively influenced older adults’ PA participation [16]. This supports our finding that the low-active group was significantly less likely to walk six blocks compared to the high-active group. In our study, the relatively low rating for environmental barriers could be attributed to the senior housing structure and programming that support PA. However, the observed differences between the low-active and high-active groups suggest that not all residents were benefiting equally.

Targeting identified barriers has the potential to produce better outcomes. To increase motivation, one might prioritize a task, such as a walking game, to encourage participation in physical activity (PA). Other low-cost motivational approaches include creating a structure that promotes social support and goal setting, providing positive messages such as “exercise is fun”, and cognitive restructuring to address negative and self-defeating attitudes and misconceptions. These strategies can enhance long-term PA engagement by boosting exercise self-efficacy, control beliefs, self-regulation, and action planning [17]. Assisting residents to prioritize PAs that have minimal opportunity costs could be most effective [18]. For example, offering on-site group exercise classes at a minimal cost or facilitating a free, peer-led group exercise program. In addition, community exercise programs can help address physical health barriers. Participation in these programs has been shown to positively influence exercise motivation, potentially mitigating the effect of health conditions [19]. In addition, some residents reported environmental barriers, but this typically requires long-term community planning. To promote walking for transportation, residents benefit from access to shops and services, well-maintained walking facilities, and a sense of familiarity and safety from crime [20].

Although it was expected that older adults are more likely to have a fall and less likely to leave the house, no significant difference in these two outcomes was found between the low-active and high-active groups. This may be because the low-active group still has some activity, stabilizing muscles to prevent falls. Their lifestyles, such as a job or social life, likely drive their need to leave the house, and would not be affected by the amount of PA in which they engage. However, the low-active group was significantly less likely to walk six blocks than the high-active group, serving as a barrier to reaching higher PA levels. Additionally, no significant differences in emotional health, skills, social, and external barriers to PA were observed between the low-active and the high-active groups. This may be because the low-active group still engages in PA, just not to the same PA levels as the high-active group. These variables may influence engagement in PA, but not specifically at moderate-to-vigorous intensities or levels of PA.

Strengths of this research include its focus on older adults in low-income senior housing, an understudied group. Limitations include participants were from only two counties in the Midwest region of the U.S., the sample size was relatively small, and self-reported measures of PA, such as the IPAQ and IPAB, are commonly biased, as they are subject to recall and social desirability bias, which can result in overreporting of PA and misinterpretation of perceived barriers. The cross-sectional design also precludes casual inference.

Further research is needed to objectively document PA and provide a more detailed description of barriers to PA in this population, possibly using a qualitative component. This could provide a better understanding of the impact of barriers on PA and elucidate the underlying factors that contribute to the PA barriers for low-active and high-active groups. Although examining the relationship between the barriers to PA and the PA variables by group was not the aim of the study, future studies should consider modeling gradients of PA behaviors for both low-active and high-active groups to expand our understanding of this issue. Ultimately, the results of this work will be used to design and test interventions that address the barriers to PA in this population. Additionally, one of the major limitations of this study is its lack of adjustment for demographics such as gender, race/ethnicity, education, marital status, and health conditions. Future studies should consider using analyses like ANCOVA to control for these covariates, which would improve the model and strengthen the results.

## 5. Conclusions

Older residents of low-income senior housing reported a wide range of PA. Residents with low PA reported significantly less time spent in all types of PA: moderate and vigorous PA, resistance training, and walking. They reported more total barriers and more physical health, environmental, and prioritization barriers but no significant differences in emotional health, skill, social, or external barriers to PA. Further research is necessary to gain a deeper understanding of the characteristics and underlying mechanisms of these barriers and to develop effective strategies for addressing them.

## Figures and Tables

**Table 1:** Description of Participants.

Variables	Low-Active Group	High-Active Group	Total Sample
	N (%)	N (%)	N (%)
Gender			
Male	12 (26.1%)	11 (23.9%)	23 (9.5%)
Female	34 (73.9%)	35 (76.1%)	69 (28.4%)
Race/ethnicity			
White	31 (67.4%)	30 (65.2%)	61 (25.1%)
Hispanic, Latino, or Spanish American			
Black or African American	4 (8.7%)	4 (8.7%)	8 (3.3%)
Asian	9 (19.6%)	8 (17.4%)	17 (7.0%)
American Indian or Alaska Native	1 (2.2%)		1 (0.4%)
Middle Eastern or North African	0 (0%)	2 (4.3%)	2 (0.8%)
Native Hawaiian or Other Pacific Islander	0 (0%)	0 (0%)	0 (0%)
Some other race or ethnicity	0 (0%)	0 (0%)	0 (0%)
Education			
Less than high school	1 (2.2%)		1 (0.4%)
Some high school	4 (8.7%)	1 (2.2%)	5 (2.1%)
High school graduate or GED	15 (32.6%)	15 (32.6%)	30 (12.3%)
Vocational training (beyond high school)	1 (2.2%)	1 (2.2%)	2 (0.8%)
Some college (less than 4 years)	9 (19.6%)	13 (28.3%)	22 (9.1%)
College/University Degree	7 (15.2%)	6 (13.0%)	13 (5.3%)
Graduate/Professional Education	5 (10.9%)	7 (15.2%)	12 (4.9%)
Marital Status			
Married	8 (17.4%)	11 (23.9%)	19 (7.8%)
Separated/Divorced	9 (19.6%)	7 (15.2%)	16 (6.6%)
Widowed	14 (30.4%)	12 (26.1%)	26 (10.7%)
Never married	9 (19.6%)	9 (19.6%)	18 (7.4%)
Not married but living with a partner	0 (0%)	0 (0%)	0 (0%)
Self-rated health			
Walks 6 blocks			
Yes = 1	28 (60.9%)	37 (80.4%)	65 (26.7)
No = 0	17 (37.0%)	6 (13.0%)	23 (9.5%)
Leaves house			
Yes = 1	35 (76.1%)	34 (73.9%)	83 (34.2%)
No = 0	10 (21.7%)	12 (26.1%)	8 (3.3%)
Experienced a fall			
Yes = 0	10 (21.7%)	12 (26.1%)	22 (9.1%)
No = 1	35 (76.1%)	34 (73.9%)	69 (28.4%)

Note: N = Number, % = Percentage.

**Table 2:** Descriptive statistics of the study outcomes by group and for the total sample.

	Total Sample (Sample Size = 92)	Low-Active (Sample Size = 46)	High-Active (Sample Size = 46)			
Variables	Mean (SD)	Mean (SD)	Mean (SD)	*t*	*df*	*p*
IPAB Emotion	1.97 (0.802)	2.10 (0.886)	1.85 (0.696)	1.505	90	0.136
Physical	2.27 (0.972)	2.50 (1.055)	2.04 (0.830)	2.329	90	0.022 *
Priority	2.38 (1.002)	2.66 (1.037)	2.09 (0.887)	2.836	90	0.006 **
Skills	2.03 (1.124)	2.13 (1.166)	1.93 (1.083)	0.834	90	0.407
External	2.25 (0.859)	2.36 (0.927)	2.14 (0.781)	1.216	90	0.227
Social	2.23 (1.036)	2.38 (1.141)	2.08 (0.907)	1.416	90	0.160
Environ	1.83 (0.897)	2.02 (1.073)	1.64 (0.631)	2.072	72.8	0.042 *
Total	2.14 (0.731)	2.31 (0.794)	1.97 (0.625)	2.281	90	0.025 *
IPAQ VPA ^a^	288.26 (658.227)	15.30 (54.594)	561.22 (849.029)	−4.352	45.4	<0.001 **
MPA ^a^	427.13 (787.872)	47.74 (95.819)	806.52 (975.614)	−5.250	45.9	<0.001 **
Walk ^a^	995.74 (4160.968)	130.13 (174.89)	1861.34 (5783.565)	−2.029	90	0.045 *
TotalPA ^a^	1711.13 (4493.524)	193.18 (220.879)	3229.08 (6006.053)	−3.426	45.12	<0.001 **

Note: * = *p* < 0.05; ** = *p* < 0.01 comparing the Low-Active group and the High-Active group. IPAB: Inventory of Physical Activity Barriers. Emotion: Emotional health barriers, Physical: Physical health barriers, Priority: PA priority barriers, Skills: Skills barriers, External: External factor barriers, Social: Social barriers, Environ: Environmental barriers, Total = Total barriers. IPAQ: International Physical Activity Questionnaire. VPA: Vigorous Physical Activity, MPA: Moderate Physical Activity, and TotalPA: Total Physical Activity. ^a^ MET-minutes per week.

## Data Availability

The data presented in the study are available on request from the corresponding author.
